# Application of workload indicators to assess the allocation of orthopedists in a national referral hospital in Brazil

**DOI:** 10.1186/s12960-021-00666-0

**Published:** 2022-01-28

**Authors:** Claudia Regina Machado, Deise Brasil, Mario Roberto Dal Poz

**Affiliations:** 1 Dept. of Anesthesiology, Medical Sciences School, University of the State of Rio de Janeiro (FCM/UERJ), Rio de Janeiro, RJ Brazil; 2 Former Consortium for Brazilian Accreditation (CBA), Rio de Janeiro, RJ Brazil; 3 Social Medicine Institute, University of the State of Rio de Janeiro (IMS/UERJ), Rio de Janeiro, RJ Brazil

**Keywords:** Health workforce allocation, Workload, Orthopedics, Specialized hospital, WISN

## Abstract

**Background:**

The study analyzes the allocation of specialized doctors’ orthopedists in a high-complex hospital, using the WHO’s Workload Indicators of Staffing Need (WISN) methodology and approach, which measures the workload pressure on the healthcare team (positive, negative, or well-adjusted).

**Methods:**

In the first phase, the hospital’s operations and activities were analyzed using the information system. The duration of the tasks performed by the specialist physicians was observed and directly measured in the second phase. Finally, the indicators were analyzed, and the workload was calculated using the WISN application. The measurement was made using the available work time per year divided by the time unit over the previous 12 months.

**Results:**

The hand surgery care unit was WISN 1.0 and the ratios for the spine surgery care unit was 1.22, indicating enough physicians and no work overload among the groups surveyed. The ratio in the knee unit was 1.69, indicating that there was an excess of staffing for the workload.

**Conclusion:**

The workload findings and staffing calculations were useful in supporting and orienting the design and implementation of measures to increase the efficiency and effectiveness of health services.

## Background

Health services administrators worldwide are facing growing challenges and insufficient resources to meet the demands in various areas of health services, and the situation is no different in Brazil [1, 2]. Health workers account for three-fourths of the recurrent budgets, and it is necessary to verify whether the workforce at a given healthcare facility is at the ideal level to produce the expected for the workload [3].

The situation does not differ greatly at orthopedic and trauma services, where there is often a shortage of both trained staff and material resources for adequate patient care. This highlights the need to reinforce and develop programs and methodologies that meet the expectations for the healthcare staff’s development and valorization, besides increasing the institution’s efficiency, a situation that has apparently been felt in a variety of institutions [4].

The healthcare network in traumatology and orthopedics in Brazil has been pressured by the rise of urban violence in metropolitan areas, altering the country’s morbidity and mortality profile. The increase in deaths and hospitalizations from external causes—accidents and violence—is reaching virtually epidemic proportions and currently represents one of the country’s main health problems [5, 6]. The growth of the elderly population also merits attention, especially considering the comorbidities in this age bracket, sometimes victims of household or traffic accidents and incapacitating diseases with prolonged evolution, such as rheumatological disorders and osteoporosis, causing serious problems with mobility [7].

Considering that the work process in health depends on workers with skills and specializations, in addition to the growing work with collective characteristics, financing, and organization, the objectives for any policy involving the workforce should prioritize the allocation of qualified staff with specific skills, where they are necessary, performing appropriate functions for their training.

The staffing and skills levels are among the variables that determine length of hospital stay and quality of the outcome for patients [3]. Cost reduction measures can have a significant impact on health systems’ quality and accessibility, and staff cuts can place patients at risk, even jeopardizing emergency treatments.

For a long time, the calculation of health units’ staffing was based on the desired proportion of staff for the population. However, this may have been appropriate in a broader context, but not for dealing with the discrepancies related to regional morbidities, level of demand, and institutional workloads. The installations in different locations may have different requirements, even with the same installed capacity, i.e., number of beds [3]. The World Health Organization (WHO) thus developed an approach to determine the appropriate healthcare staffing levels, considering the common routine activities, time needed to perform certain activities, the activities associated with the job description, annual available working time, and the annual statistics pertaining to the volume of work. The WHO method is called the Workload Indicators of Staffing Need (WISN) [8].

The study of the quantitative and qualitative composition of healthcare staff in each institution based on workload indicators, such as the Workload Indicators of Staffing Need (WISN) as recommended by the WHO, is one of the options for supporting and orienting initiatives for improvements in both quality and safety and the number of patients to be covered with adequate treatment.

Workload assessment allows supporting decision-making in various situations to improve the existing staffing, based on priority-setting in the allocation of new workers or their redistribution or that of their activities, depending on each given service’s priority. The assessment can also contribute to the evaluation of the number of existing professional categories and the possible elimination or addition of a new category, through a review of the job responsibilities and observation of possible overlapping of tasks. This methodology allows comparison of work performance between units or departments by determining work standards for the performance of certain activities, as well as planning future needs using data on the workload in the planned services and the impact of different employment modalities on staffing need, including changes in contract formats, vacation leave, or the introduction of training policies.

The WISN method has been applied by the World Health Organization (WHO) and collaborators in various countries [9–11], including Brasil [12, 13], presenting results that contribute to improving health services supply.

However, a systematic review [14] showed that most of the studies on staffing are focused on nursing. The other health professions are under-represented and have been studied less. Another relevant point is the small number of publications on physicians, even though some regions suffer a shortage of them. In addition, even when the numbers appear adequate, not all the workers are trained to perform the necessary procedures and techniques.

The few studies that address all the health professions are related to primary care. All the studies that used the Workload Indicators for Staffing Need clearly showed that it is a tool for allocating health workforce at various levels of complexity, with the intention of getting a more balanced distribution of human resources because of the analysis. The review did not identify any references to the method’s previous use in high-complexity services or in orthopedics [14].

The current study thus aimed to contribute to improving decision-making processes in health workforce management and allocation of specialized orthopedic doctors, as well as to experiment a methodology for calculating and analyzing workload indicators in a highly complex orthopedic and traumatology healthcare unit to estimate the staffing needs of these specialists.

Following the WISN method and approach, a study was performed in a national reference hospital for traumatology and orthopedics in Brazil, analyzing the tasks and allocation of orthopedists in their wards, care units, and surgical center while conducting complex procedures.

Patient care in traumatology and orthopedics in Brazil and particularly in Rio de Janeiro is increasingly pressured by the rise in urban violence in metropolitan areas and by the Brazilian population’s progressive aging, which has modified the country’s morbidity and mortality. Meanwhile, the system faces difficulty providing adequate and timely responses to care for the population’s demands.

A burden-of-disease study using accumulated knowledge from various areas to quantify population mortality and morbidity data, collected systematically, and processed methodologically to provide important backing for health policies, revealed external causes as the second leading overall cause in Brazil and the third in the country’s Southeast region in terms of disability-adjusted life years [15].

The current study was performed in a large high-complexity hospital in orthopedics and traumatology, with 38 consultation offices, 255 in-patient beds in the wards, 48 intensive care beds, and 21 operating rooms. In 2013, the out-patient productivity was 208 217 treatments, 17 351 medical consultations, and 9959 surgeries. The hospital mortality rate is low, only 0.48%, and mean length of stay was 7.6 days.

In its role as a specialized referral center, the hospital has experienced difficulties in providing adequate and timely responses to fully meet the population’s demands. Patients thus often make repeated attempts to receive treatment and suffer postponements and inadequate care, causing exacerbation of the cases and an increase in the number of sequelae, resulting in demands for high-complexity procedures. Such factors are associated with low linkage between health units, requiring more effective intervention and implying a more comprehensive solution. With the formation of a Trauma Network, the responses to this complex problem were expected to achieve greater case-resolution capacity and thus fewer high-complexity procedures and sequelae [16].

In 2006, the hospital’s administration created Specialized Care Centers, aimed at improving the quality of care provided, making it more comprehensive through a line-of-care approach. The Specialized Care Units consist of healthcare professionals from various fields: physicians, nurses, social workers, psychologists, and in some cases physical therapists.

Starting in 2011, there was a significant increase in all the surgical procedures in the specialties, featuring knee surgery with a 57% increase, while the spine group and hand group witnessed increases of 43% and 48%, respectively, during the same period. This increase was due to greater installed capacity, which allowed offering the different sub-specialty groups more operating rooms per week.

The data showed an increase in the numbers of both outpatient consultations and surgical procedures starting in 2011. Several factors may explain this increase, such as an increase in the installed capacity, increase in the number of medical components in the Specialized Care Centers, better management of the activities in the centers, and the growing demand for more complex procedures (Table 1). These data were collected through reports generated by computerized system using the electronic medical record records.

**Table 1:** Surgical and outpatient production, orthopedics and traumatology hospital, Brazil, 2011–2014

Surgical and outpatient production	2011	2012	2013	Up to Sept./2014
Surgeries performed	5662	6784	9659	5114
High-complexity surgeries (%)	35.16%	34.83%	34.19%	
Outpatient treatments	155 227	177 161	208 217	125 830
Treatments	122 470	146 742	180 994	
Orthopedic and surgical	53 326	60 967	76 287	55 041
Complementary	22 270	21 972	28 030	16 756
Multidisciplinary	46 757	63 524	76 479	53 594
Other	117	279	198	439
Triage	15 043	13 107		
Hospital admissions	5761	7023	9830	5362
Home visits	11 953	10 289	17 393	9348

Source: Data from the hospital’s information system

**Fig. 1:**
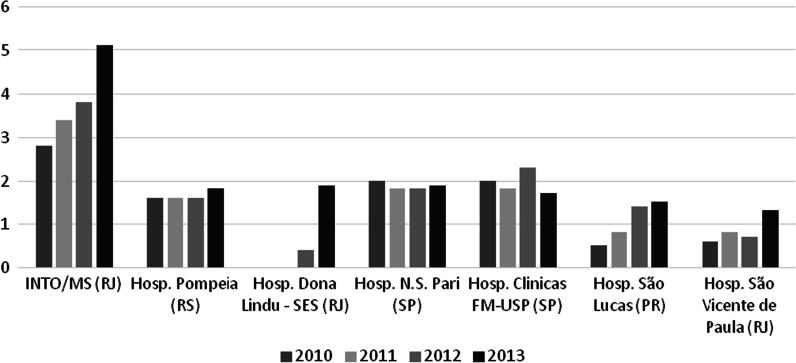
Percentage share of high-complexity procedures in orthopedics per hospital, 2010–2013, Brazil (Source: prepared by the authors)

The hospital also performs most of the most prevalent surgeries in the state and city of Rio de Janeiro, concentrating most surgical procedures in orthopedics and traumatology (Fig. 1).

When comparing hospital data with records from the Ministry of Health’s Department of Informatics of the Brazilian National Health System (DATASUS), this system contains information that allows the generation of reports based on a registry of all health establishments that are part of the Unified Health System (SUS). Among the outputs, there are outpatient and inpatient information reports. It becomes clear that the most common procedures in each group at the regional and national levels are those performed by the institution (knee: primary arthroplasty; spine: thoracolumbar arthrodesis; and hand: peripheral nerve neurolysis).

## Methods

The methodology was developed in two stages. The first stage focused on the hospital’s functioning, using information from the computer system, and directly recording the time spent in each activity using semi-structured scripts over the course of 4 weeks. The second stage involved the application of the computer-based Workload Indicators of Staffing Need (WISN) for calculating the standard workload, which is equal to the annual available working time divided by the unit of time. The information obtained from the institution’s computer system dates from 2013.

The workload components are represented by the patient care service and support activities, for example.

The phases of data collection were as follows:

- Revision and adaptation of the data collection instrument for the high-complexity unit, aimed at testing the method and potentially validating it.
- Data collection on the number of professionals and their training (mapping).
- Evaluation of retrospective data in the institution’s computer systems, through research related to duration of consultations, surgeries.
- Direct observation of the time spent on each activity in predetermined time intervals for 4 weeks, such as outpatient consultation time, surgery time, dressing.
- Evaluation of the factors impacting the workload (overload of the physicians, potentially causing sick leave due to physical or mental health problems and affecting patient safety); and
- Discussion of the results achieved with the physicians involved to validate the collected data and identify the strengths and weaknesses and possible improvements to the process.

The method for estimating the necessary time were identified through interviews, consultation of specialists’ opinions, and observation and annotation of the time spent on the activities, which required instructing some assistant nurses to take the responsibility for the recording process.

Outpatient data were obtained through direct observation, once a week for 4 weeks.

The results obtained with recording the time on orthopedists’ work activity in the outpatient setting were achieved by direct observation by the same observer once a week for 4 weeks, and later validated by these professionals in meetings with the presentation of the findings during the consultations.

Direct observation of the time spent in surgical procedures was done continuously over the course of 4 weeks. For operational reasons, this task was performed by nurse technicians in the operating theater itself, in different shifts. The collected data were like the numbers recorded in the hospital’s computer system.

It is worth pointing out that the average time for carrying out these surgical procedures does not correspond exactly to the time for using the operating rooms, which obviously includes the time for cleaning and disinfecting them, as well as the time for preparing and undertaking anesthetic procedures. Depending on the type of surgery performed, these activities have routines and guidelines, so that they are performed in a uniform manner.

Following that, both the data obtained through the hospital information system in 2013 and the data collected through direct observation over a 4-week period in 2014 were imported into the WISN program to obtain the results in this phase, significantly facilitating the process. The target professional categories were recorded in the application, in this case orthopedists specialized in spine, knee, and hand, informing the weekly workload, holidays, vacation, sick leaves, and training courses.

Before starting the fieldwork, the study was submitted and approved by the Institutional Review Board (IRB) and Ethics Committee. Participants were informed of the study’s objectives and signed a free and informed consent form.

## Results

The available working time indicates the weekly hours and days of work and the number of leaves per year for each professional category at the unit. In the specific case, the imputed information was for 38 weeks, with 76 days worked, corresponding to 762 h (Table 2).

**Table 2:** Available working time

Staffing indicators	Orthopedists, spine	Orthopedists, knee	Orthopedists, hand
Weekly days of work	2	2	2
Daily hours of work	10	10	10
Annual leave			
Holidays	15	15	15
Sick leave			
Special leave without notice			
Training days per year	12.75	12.75	12.75
Days not worked	27.75	27.75	27.75
Weeks not worked	13.88	13.88	13.88
Weeks of work	38.12	38.12	38.12
Days of work	76.24	76.24	76.24
Number of hours	762.4	762.4	762.4

Source: Prepared by the authors using the WISN methodology

In the results of applying the WISN methodology, the measure of workload pressure on medical staff is the ratio of the actual to the required number of physicians, presented as positive, negative, or well-adjusted.

If the ratio between these two numbers is close to one (~ 1), the current staffing is balanced with the health unit’s workload demands; a ratio greater than one (> 1) indicates surplus staffing in relation to the workload, while a ratio less than one (< 1) indicates that the current staff is insufficient to cover the workload. Therefore, the lower the ratio, the higher the workload pressure.

In the comparison of staffing in the three target groups, both the hand and spine care units showed WISN ratios of approximately 1, meaning adequate staffing and absence of work overload (Table 3). Meanwhile, the ratio was higher than 1 in the knee center, revealing surplus staffing in relation to the center’s surgical output.

**Table 3:** Staffing comparison: presents the difference between the existing staff numbers and WISN ratio in the target hospital

STAFF	A Existing staff	B Calculated need	C Staff difference	D WISN ratio (A/B)
Orthopedists, spine	8	7	1.4	1.2
Orthopedists, knee	19	11	7.7	1.6
Orthopedists, hand	13	13	0.3	1.0

Source: Prepared by the authors using the WISN methodology

The spine center only presents WISN greater than 1 for cases of follow-up consultations. The analysis of the statistics for the spine group’s workload showed the need for four more physicians to perform the follow-up consultations.

The same procedure was used for the hand and knee groups.

The hand group showed a WISN ratio of 1, but it showed a staffing surplus both for surgeries and for performing dressings, performed in post-operative visits. This ratio represents the activity standards. A WISN ratio of 1 means that the workload is balanced or adequate. In 2013, the hand group had 15 physicians with 20-h workweeks and two physicians with 40-h weeks.

In the knee group, the system showed WISN ratios greater than 1 both for surgeries and for follow-up visits on the ward, which means a staffing surplus.

## Discussion

The spine group’s low case-resolution capacity stands out, which may be due to numerous factors, such as the scarcity of specialized surgeons in this area, the long training time to prepare these specialists for complex procedures, dependence on external factors, such as the availability of blood and blood products, and vacancies in intensive care, besides the long operating time for spinal surgical procedures.

The spine group in 2013 had eight surgeons on 20-h workweeks and three on 40-h weeks, all of whom are still at the service, and the data for these 11 surgeons were used. One of these staff surgeons conducted his work in four operating rooms simultaneously, through supervision of residents during our 4 weeks of direct observation, so this surgeon’s total operating time in minutes was far greater than that of his colleagues.

When the results were presented to the group of surgeons for validation, there were questions about the need to eliminate the statistics for this surgeon. This would not have been feasible, and the result would not have been trustworthy, since the system assesses the group as a whole and these supervised surgeries by residents took place.

Despite the small proportional number of spinal surgeries, they also correspond to non-immediate post-operative follow-up, requiring longer evaluation, besides the large number of patients that need to be evaluated in a long waiting list for surgery.

Some questions need to be analyzed in detail in the situation of knee group of surgeons, either because not all these physicians were fully prepared to perform the surgeries on their own (because they were still in training), there had been a physicians’ strike, the blood bank was experiencing shortages, or other logistic negative factors.

In relation to workload, certain activities are determinant in each type of health unit, such as the numbers of surgeries, hospitalized patients, laboratory tests, and teaching and training sessions, among others.

Factors that could impact workload in high-complexity units in orthopedics and traumatology include the number of beds, existence of an emergency department and day hospital, and number of operating rooms, among others.

As reported in most studies on the subject [14], it is essential in the use of the WISN method for the activity standards to be defined by consensus between administrators and health workers.

It is thus necessary to examine both the staffing differences and the ratios, using the knowledge of the local situation to interpret what these numbers mean. Importantly, this approach does not rule out administrative factors, as can occur with the poor distribution of tasks among the various workers or the use of medical residents under supervision.

Following the steps proposed by the World Health Organization [8], the WISN method has been applied by the WHO itself and its collaborators in various countries, yielding results that have contributed to improving health services supply [17].

The current study’s results were validated by the physicians involved, lending legitimacy to the outcome. Occasional discrepancies in the results may reflect staff who were working at tasks outside their job role or factors preventing them from performing their work adequately and safely.

The results show that there is no shortage of physicians in the three groups studied. It is thus crucial to raise some questions on the surgical output versus patients’ waiting line, since the WISN ratio did not reveal a work overload in any of the three groups.

According to the results, the number of patients joining the waiting line exceeded the number of surgeries performed, which may be explained by the fact that the surgeries are subdivided by procedures (“sub-lines”). Example: primary knee arthroplasty, etc. (elective and non-emergency procedures). Thus, not all the sub-lines have the same number of surgeries performed in relation to the number of patients entering the waiting line.

The surgeries performed do not originate only from the components of the sub-lines, but also include reinterventions (second look) due to complications such as infection and prosthesis luxation, among others, plus emergencies referred via SISREG (surgical dashboard), which require quicker intervention, since they result from acute trauma.

## Conclusions

This study validated both the method and the software developed by the WHO for calculating workload indicators for staffing needs, in this case in a complex health unit.

The WISN tool had only been validated previously for primary care, in a very few countries, supporting the setting up of health teams, so we believe that the current study is highly relevant and are convinced that it can be used by other institutions to orient human resources management measures and potentially to improve the method itself.

It can also be used as a management monitoring tool, with the study being repeated every 1 or 2 years with the aim of enhancing the distribution of the institution’s human resources.

It is worth noting that the application (software), despite making the calculations accurately, cannot incorporate all the peculiarities of the institutions and health services and in which it is applied.

In this context, it is necessary to assess the possibilities for improving the management of the waiting line, such as problems with excessive waiting time in some cases; lack of clear criteria for calling patients for tests and surgery, among others. Such improvements require greater efficiency in the operating processes, mainly in the patient’s preparation for surgery and in the mechanism for revising waiting lines, to obtain a real and up-to-date diagnosis, besides adequate planning in scheduling surgical procedures.

Several measures have been discussed to reduce the problems that were identified, particularly investments in management training and training to increase quality and safety for the hospital’s employees. This effort is expected to bring positive consequences in the medium term.

One of the method’s shortcomings is that it doesn’t consider the impact of external factors including blood and blood product shortages in surgical procedures, as well as data incompleteness or inaccuracy, which can lead to inconsistent outcomes and incorrect recommendations [13].

The study’s limitations include the fact that it was based on data from 2013; since then, various technical and administrative measures have been taken to modify this situation, such as a change in the department head and analysis and management of the waiting line, for example.

The method’s applicability should consider planning and its strategies, a well-defined purpose, and integration into the management system. It should also generate questions such as which hospital departments have surplus staffing or staff shortages, and which final data need to be validated.

Since this is the first study to use the method to record time in highly complex activities in orthopedics and traumatology, there is no way to benchmark with other institutions to assess whether the activity times could be longer or shorter, or whether some failure is occurring, for example, by not performing some step in the care process. Thus, we believe that other studies, including similar institutions, are necessary to make recommendations about the ideal times for some activities to reach more solid and trustworthy conclusions and recommendations.

However, WISN is clearly a useful tool, well-suited for calculating staffing need in complex health units.

This has longed be proved that the WISN method is an objective way of establishing staffing levels, but it requires a team with adequate knowledge to make the crude data meaningful for the calculations and their results [3].

## Data Availability

The data sets used and/or analyzed during the current study are available from the corresponding author on reasonable request.

## Abbreviations

WHO: World Health Organization
WISN: Workload Indicators of Staffing Need
IRB: Institutional Review Board
DATASUS: Department of Informatics of the Brazilian National Health System
SUS: Unique Health System
